# Olfactory Groove Meningioma Compressing the Optic Chiasm and Optic Nerve: Improvement of Visual Functions by Surgical Intervention

**DOI:** 10.22336/rjo.2025.39

**Published:** 2025

**Authors:** Sevgi Tongal, Ismail Uyanik, Fatma Poslu Karademir, Ihsan Cakir, Asli Inal

**Affiliations:** Department of Ophthalmology, Istanbul Beyoglu Eye Training and Research Hospital, University of Health Sciences, Istanbul, Turkey

**Keywords:** olfactory groove meningioma, visual loss, ophthalmology, optic chiasm, visual field, OGM = Olfactory Groove Meningioma, VF = Visual field, RNFL = Retinal nerve fibre layer, MRI = Magnetic resonance imaging, VA = Visual acuity, OCT = Optical coherence tomography, RAPD = Relative afferent pupillary defect, VFI = Visual field index, MD = Mean deviation, PSD = Pattern standard deviation, cf = counting fingers, OD = Optic discs

## Abstract

**Objective:**

Olfactory groove meningiomas (OGMs) are rare but serious tumours that can cause visual loss. Surgical treatment of such meningiomas is critical for the recovery of visual functions.

**Methods:**

In this case report, a 62-year-old patient presented to our clinic with sudden visual loss in the right eye and underwent ophthalmological examination. Visual field (VF), retinal nerve fibre layer (RNFL) thickness test, and contrast-enhanced cranial and orbital magnetic resonance imaging (MRI) were performed. The patient was diagnosed with OGM compressing the optic chiasm and nerve, and was operated on by the neurosurgery department. Post-operative examinations were repeated, and the patient was followed up regularly.

**Results:**

Evaluations revealed a significant improvement in the patient’s visual functions, including visual acuity and visual field (VF). Postoperative RNFL-thickness tests showed minimum variation. Early surgical treatment of OGMs compressing the optic chiasm and the optic nerve resulted in significant improvements in visual acuity and visual field (VF).

**Discussion:**

This case demonstrates that early surgical intervention in olfactory groove meningiomas can result in substantial improvements in visual acuity and visual fields, regardless of the initial severity of visual impairment. Even in cases with profoundly reduced vision, significant functional recovery is possible following timely decompression. Preoperative preservation of retinal nerve fiber layer (RNFL) thickness may serve as a favorable prognostic indicator for postoperative visual outcomes. The integration of MRI, VF, and OCT findings provides a comprehensive framework for diagnosis, treatment planning, and follow-up.

**Conclusions:**

According to the literature, surgical intervention is efficacious in improving visual functions and emphasizes the importance of early surgical treatment for such cases.

## Introduction

Olfactory groove meningiomas (OGMs) account for approximately 10% of all intracranial meningiomas and 2% of all intracranial tumours [1]. These tumours typically develop in the midline of the anterior cranial fossa and are most common in adults, peaking in the 5th decade of life [2]. Despite their tendency for slow growth, OGM can expand bilaterally in an asymmetric manner and may go unnoticed until clinical symptoms emerge due to their gradual progression. The most common presenting symptoms include anosmia, headache, personality changes, and visual disturbances, with vision loss often being a late manifestation of tumor compression on the optic nerve or optic chiasm [3]. Surgical resection remains the primary treatment modality, with total tumor removal achievable in 67-100% of cases [4].

OGM tumours can cause significant morbidity, particularly when they exert pressure on the frontal lobes and visual pathways, leading to visual impairments [5]. Being near the tumor, the optic chiasm is especially vulnerable to compression, resulting in visual field deficits, most commonly bitemporal hemianopia. However, unilateral visual loss can also occur depending on the specific pattern of tumor growth and nerve compression [6]. A crucial predictor of postoperative visual recovery in patients with parachiasmal meningioma is the thickness of the retinal nerve fibre layer (RNFL) [7]. It is a vital characteristic to look at in these situations because it indicates the vitality of the structural nerve fibre axons. Early and thorough assessment is critical because morphologic abnormalities in the optic disc appearance and RNFL often precede visual field loss in diseases such as OGM and glaucoma [8].

In this report, we present a rare case of an OGM causing acute unilateral vision loss due to optic chiasm and optic nerve compression, successfully treated with surgical resection. Given the dramatic improvement in visual acuity (VA) and visual field (VF) following the surgical procedure, we hope that highlighting the importance of early diagnosis and treatment will shed light on similar cases.

## Materials and methods

### Case Presentation

A 62-year-old female patient who applied to the clinic due to a sudden vision loss in her right eye underwent a series of ophthalmologic examinations, imaging studies, and surgical treatment over 18 months. A written consent was obtained from the patient for the study. Ethical approval is not required for the case report.

### Patient examination

**Fig. 1:**
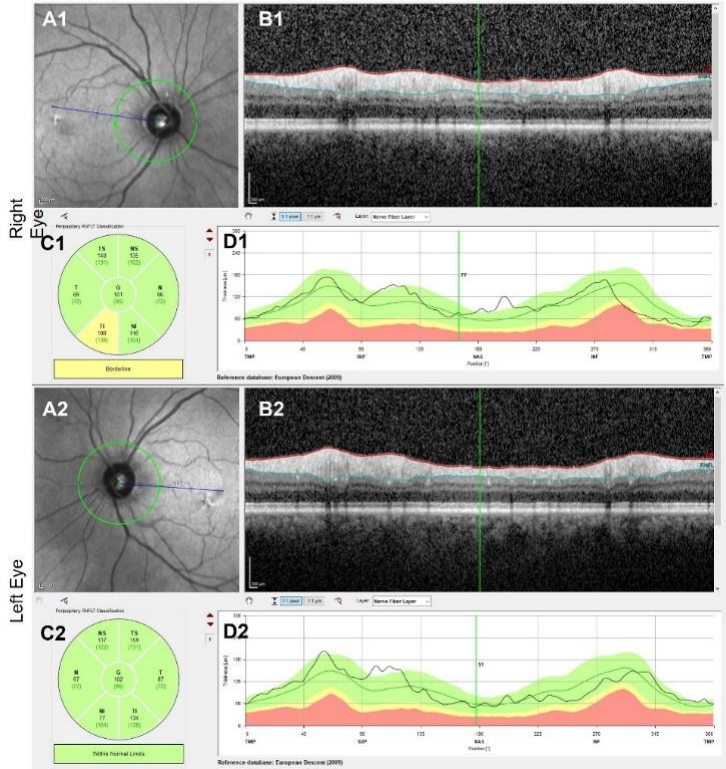
Pre-operative optical coherence tomography (OCT) measurement of Retinal Nerve Fibre Layer (RNFL) thickness of both eyes (A1 and A2). RNFL thickness measured along the circumference of the 3.5 mm circle centred on the optic nerve head (B1 and B2). Circumferential OCT scan of the peripapillary retina along the circle depicted in A1, A2, C1, and C2. Quantification of peripapillary RNFL thickness by sector (D1 and D2). Quantification of RNFL thickness plotted against the position along the peripapillary OCT scan (in degrees)

Before surgery, the patient underwent further ophthalmologic evaluations to document the progression of her condition. Visual acuity (VA) testing was performed for both eyes using the Snellen chart. The pupillary reflex examination, assessed by the swinging light test, revealed a positive relative afferent pupillary defect (RAPD) in the right eye. Retinal nerve fibre layer (RNFL) thickness was measured using spectral-domain optical coherence tomography (OCT) (Heidelberg Engineering, Germany), revealing stable RNFL thickness of 101 μm in the right eye and 102 μm in the left eye (Fig. 1). Visual field (VF) testing, performed with the Humphrey Visual Field Analyzer (30-2 test pattern, Carl Zeiss Meditec, USA), confirmed severe VF loss (generalized loss) in the right eye. The left eye displayed milder VF defects with some frame loss. However, visual acuity and RNFL thickness in the left eye remained unaffected.

**Fig. 2:**
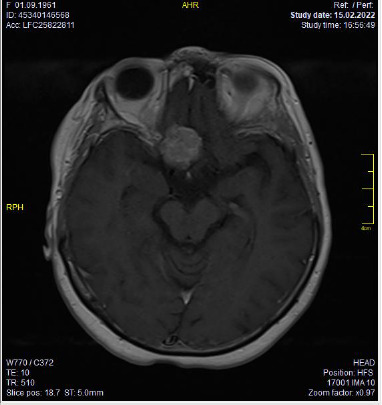
A contrast-enhanced magnetic resonance imaging (MRI) of the brain and orbits of the patient

To investigate the cause of the patient’s sudden vision loss, contrast-enhanced magnetic resonance imaging (MRI) of the brain and orbits was performed (Fig. 2).

### Surgical Intervention

The patient underwent a surgical resection of the OGM. The neurosurgical team performed a craniotomy to remove the tumor, to relieve pressure on the optic chiasm and nerve, and restore visual function. The surgery was successful in decompressing the optic pathways.

### Post-operative follow-up

The patient was closely monitored after surgery, with follow-up ophthalmologic examinations conducted at regular intervals to assess visual function and the health of the optic nerve. The VA testing, pupillary reflex examination, and VF testing were performed during post-operative visits. OCT was repeated to monitor changes in RNFL thickness.

### Long-term follow-up

The patient was re-evaluated on several occasions after surgery to ensure the stability of her visual function at 6-month intervals.

## Results

**Fig. 3:**
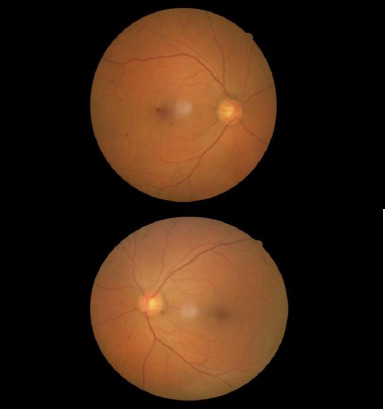
The fundus image of both eyes was normal

The patient presented with sudden vision loss in the right eye. Examination revealed counting fingers vision at 10 cm in the right eye and 20/20 Snellen acuity in the left eye. The right pupil was fixed, mid-dilated, and exhibited a relative afferent pupillary defect (RAPD). Slit-lamp examination was normal. Intraocular pressure measured by applanation was 14 mmHg in both eyes. Dilated fundus examination was normal, and both optic disc borders appeared sharp (Fig. 3). Initial RNFL thickness was 101 μm in the right eye and 102 μm in the left (Fig. 1). MRI revealed a 29x19x23 mm mass at the olfactory sulcus compressing the right optic chiasm and nerve, consistent with a meningioma.

VF testing revealed a generalized loss in the right eye, with a visual field index (VFI) of 8%, mean deviation (MD) of -28.48, and PSD of 7.18. The left eye exhibited a frame defect, with a visual field index (VFI) of 87%, a mean deviation (MD) of -7.45, and a pattern standard deviation (PSD) of 7.79. OCT showed RNFL thickness values of 125 μm in the right eye and 106 μm in the left.

### Post-operative examination

The patient underwent surgery for the meningioma. Shortly after surgery, her VA had improved slightly to 20/30 Snellen acuity in the right eye and 20/25 Snellen acuity in the left eye (Table 1). Pupillary reflex examination revealed a fixed, mid-dilated pupil pre-operatively, in the right eye, and a RAPD on the right side, indicative of significant optic nerve damage. Postoperatively, the right-sided RAPD resolved, indicating partial recovery in optic nerve function. Mean RNFL thickness was measured at 95 μm in the right eye and 98 μm in the left eye. VF testing revealed a significant improvement in the right eye, with a VFI of 98%, a mean deviation (MD) of -4.10, and a peak-to-saddle distance (PSD) of 6.08. The left eye showed a VFI of 96%, a mean deviation (MD) of -4.50, and a posterior subcapsular lenticular opacity (PSD) of 4.40. These improvements indicated effective decompression of the optic chiasm and nerve (Table 1).

**Table 1:** A summary table for initial, pre-operative, and post-operative ophthalmologic evaluation results for the right eye (R) and left eye (L)

	Right Eye				Left Eye			
Parameters	Initial (R)	Post-Op 1 (R)	Post-Op 2 (R)	Follow-Up (R)	Initial (L)	Post-Op 1 (L)	Post-Op 2(L)	Follow-Up (L)
VA	10 cm cf	20/30 20/40	20/25	20/25	20/20	20/25	20/30 20/40	20/25
OD	Vital, Sharp borders	Vital, Sharp borders	Vital Sharp borders	Vital, sharp borders	Vital, Sharp borders	Vital, Sharp borders	Vital, Sharp borders	Vital, Sharp borders
RAPD	+	-	-	-	-	-	-	-
RNFL Thickness (μm)	101	95	99	85	102	98	107	101
VF	Generalized loss	Normal	Normal	Normal	Frame defect	Normal	Normal	Normal
VFI	8%	98%	96%	96%	87%	96%	98%	98%
MD	-28.48	-4.10	-3.23	-4.10	-7.45	-4.50	-3.17	-3.17
PSD	7.18	6.08	1.83	6.08	7.79	4.40	1.91	1.91

VA = Visual acuity, cm = centimetres, cf = counting fingers, OD = Optic discs, RAPD = Relative afferent pupillary defect, VF = Visual field, VFI = Visual field index, RNFL = Retinal nerve fibre layer, MD = Mean deviation, PSD = Pattern standard deviation

### Post-operative follow-up

**Fig. 4:**
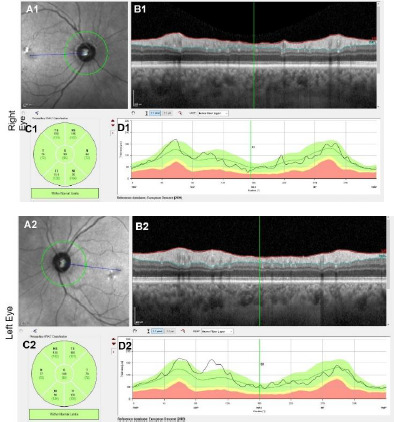
Post-operative optical coherence tomography (OCT) measurement of Retinal Nerve Fibre Layer (RNFL) thickness of both eyes (A1 and A2). RNFL thickness measured along the circumference of the 3.5 mm circle centred on the optic nerve head (B1 and B2). Circumferential OCT scan of the peripapillary retina along the circle depicted in A1, A2, C1, and C2. Quantification of peripapillary RNFL thickness by sector (D1 and D2). Quantification of RNFL thickness plotted against the position along the peripapillary OCT scan (in degrees)

By September 2022, the patient’s visual acuity (VA) was stable at 20/25 Snellen acuity in both eyes. Mean RNFL thickness was 99 μm in the right eye and 107 μm in the left. VF results showed further improvement, with a VFI of 96% in the right eye (MD: -3.23, PSD: 1.83) and 98% in the left eye (MD: -3.17, PSD: 1.91) (Fig. 4). Optic disc margins were sharp and vital in both eyes. At the follow-up in March 2023, the patient’s visual acuity (VA) remained stable at 20/30 Snellen acuity in the right eye and 20/25 Snellen acuity in the left eye. The mean RNFL thickness was 92 μm in the right eye, indicating mild thinning compared to preoperative values. In contrast, the left eye remained stable at 102 μm, and the optic disc borders remained sharp (Table 1).

### Visual field analysis

**Fig. 5:**
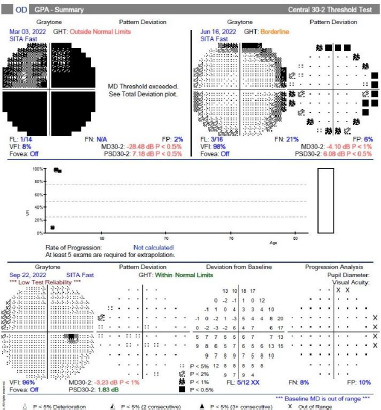
Visual field index (VFI) improvement in the right eye. A significant improvement was observed in preoperative VFI, with an 8% increase, a mean difference (MD) of -28.48, and a postoperative VFI of 98%, a MD of -4.10, and a PSD of 6.08 over 4 months

**Fig. 6:**
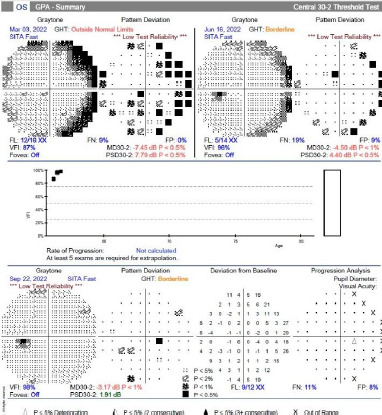
Visual field index (VFI) improvement in the left eye. An improvement was observed in the preoperative VFI (87%), MD (-7.45), and PSD (7.79) compared to the postoperative VFI (96%), MD (-4.50), and PSD (4.40) over 4 months

The patient’s VF in the right eye improved dramatically after surgery, from a generalized loss (VFI: 8%, MD: -28.48) pre-operatively to near-normal values (VFI: 98%, MD: -4.10) post-operatively (Fig. 5). The left eye, which had shown a frame defect pre-operatively, also showed recovery with a VFI of 98% post-operatively (Fig. 6). These improvements were maintained at follow-up visits (Table 1).

### Final follow-up

At the most recent follow-up in August 2023, the patient’s visual acuity (VA) remained at 20/25 Snellen acuity in both eyes. The mean RNFL thickness was measured at 85 μm in the right eye and 101 μm in the left eye (Fig. 4). Both optic discs were vital with sharp borders, and no further deterioration was noted in VFs. Therefore, thinning in RNFL thickness was within expected limits following optic chiasm and nerve compression, as well as surgery, with no significant loss of functional vision post-operatively.

## Discussion

OGMs often present with non-specific symptoms such as headaches, anosmia, or personality changes [9]. In this case, the patient’s primary symptom was acute unilateral vision loss without associated neurological deficits. OGMs can grow significantly due to their anatomical location, often causing subtle psychiatric or cognitive changes before visual symptoms appear. Visual impairment occurs when the tumor extends posteriorly, compressing the optic nerves or chiasm [10]. This compression can lead to optic disc oedema or atrophy, with the nature of VF defects depending on the tumour’s impact. While bitemporal hemianopia is the typical defect from chiasmal compression [11], this patient experienced severe right optic chiasm and nerve compression, resulting in near-total vision loss in the right eye, while the left remained largely unaffected.

RNFL thickness analysis has emerged as a valuable diagnostic and prognostic tool in compressive optic neuropathies, including optic nerve glaucomas (OGMs) [12]. Pre-operative RNFL measurements in this case were preserved, suggesting viable optic nerve fibres and correlating with post-operative recovery. Literature indicates that intact RNFL thickness before surgery predicts a greater likelihood of visual recovery [**13-15**]. Post-operative RNFL values in this patient remained relatively stable, further supporting a favourable prognosis. Conversely, significant pre-operative thinning in RNFL may indicate poorer recovery potential and necessitate aggressive treatment [16].

VF testing is essential for diagnosing and monitoring compressive optic neuropathies. Pre-operatively, this patient’s VF showed a generalized loss in the right eye, emphasizing the severity of the compression. Post-operatively, VF improvement highlighted the effectiveness of surgical decompression, with recovery in both central and peripheral vision. Such findings align with reports that early intervention can significantly restore optic nerve function and visual fields [13].

The case emphasizes the importance of early diagnosis and treatment. The patient presented with unilateral vision loss caused by a 29x19x23 mm tumor compressing the optic chiasm and nerve, confirmed via cranial and orbital MRI [17]. MRI findings, combined with VF and RNFL assessments, provided a comprehensive understanding of the tumour’s structural and functional impact, guiding surgical planning and monitoring recovery. Literature supports this multimodal approach, as MRI provides structural details while VF and RNFL analyses offer critical structural and functional insights [16].

Surgical intervention led to significant improvements in VA and VF. Pre-operatively, the patient’s right eye visual field (VF) was severely impaired (VFI: 8%), but post-operatively, it improved dramatically (VFI: 98%). RNFL thickness also stabilized after surgery. By the final follow-up, mild RNFL thinning (85 μm) was observed, a common occurrence after prolonged compression. However, this did not lead to further VF deterioration, highlighting the efficacy of timely surgical decompression [15].

This case demonstrates that early surgical intervention in OGMs can preserve visual function and prevent irreversible damage to the eye. In cases of unexplained vision loss, cranial and orbital MRI is critical for accurate diagnosis, particularly when ophthalmologic findings are inconclusive. Combining imaging data with VF and RNFL measurements offers a detailed picture of the tumour’s impact, enabling timely and effective treatment [17].

## Conclusion

In conclusion, early detection and intervention are vital in managing OGMs. RNFL and VF testing provide invaluable diagnostic and prognostic information, aiding in surgical planning and post-operative monitoring. This case highlights the importance of integrating structural and functional assessments to optimize outcomes, in line with the existing literature, which suggests that early surgical management significantly improves visual prognosis in compressive optic neuropathies.

## Data Availability

The data that support the findings of this study are openly available in this manuscript.
